# Silicosis in the Setting of Chronic Granite Exposure: A Case Report

**DOI:** 10.7759/cureus.83095

**Published:** 2025-04-27

**Authors:** Sylvia Li, Henrik Ghantarchyan, Sarkis Arabian

**Affiliations:** 1 Internal Medicine, Arrowhead Regional Medical Center, Colton, USA; 2 Pulmonology, Arrowhead Regional Medical Center, Colton, USA; 3 Critical Care, Arrowhead Regional Medical Center, Colton, USA

**Keywords:** occupational exposure to silica, pneumoconiosis, pulmonary silicosis, silica worker, silicosis

## Abstract

Silicosis develops when inhaled silica particles reach the alveoli, causing interstitial lung disease. We present the case of a 50-year-old male with no known past medical history who arrived at the emergency department with a 10-month history of dyspnea, which had worsened over the past four days. He reported working in a granite factory for 18 years, consistently wearing a mask while cutting and grinding stone. Initial laboratory tests were unremarkable. A viral respiratory panel and acid-fast bacilli cultures and smears (repeated three times) were all negative, lowering the suspicion for tuberculosis. Antifungal testing was also negative, except for a positive *Histoplasma* antibody; however, the urine *Histoplasma* antigen was negative. Bronchoalveolar lavage revealed an unremarkable cell differential, and respiratory cultures were negative. Given the negative infectious workup, along with the radiographic findings and occupational history, the patient’s presentation was most consistent with silicosis.

## Introduction

Silicosis, first described by pulmonologist Achille Visconti in 1870, is a progressive interstitial lung disease caused by the inhalation of silica particles [1]. Among the various forms of silica, the crystalline types, such as silicon dioxide, are the most pathogenic [2]. Silicon and oxygen are two of the most abundant elements in the Earth’s crust, accounting for nearly 85% of its composition. When silica particles measuring 1-3 µm are inhaled, they can penetrate the alveoli and contribute to the development of interstitial lung disease, including silicosis [3]. The duration and intensity of silica exposure correlate directly with the severity of the disease. Silicosis is commonly seen in workers involved in mining, quarrying, stone cutting, construction, glass and artificial stone manufacturing, sandblasting, cement production, and even agriculture [2,3].

Silicosis is categorized into three subtypes: acute, accelerated, and chronic. Acute silicosis, the most severe form, develops within five years of high-level exposure. Accelerated silicosis occurs within 10 years of low to moderate exposure. Chronic silicosis, as observed in our patient, typically arises after more than 10 years of low to moderate exposure to silica [2].

Diagnosis is based on a combination of radiographic findings, clinical symptoms, and occupational history [1,4]. Histologically, silicosis is marked by the presence of hyalinized and fibrotic pulmonary nodules, lymphocyte accumulation, alveolar macrophage infiltration, and thickening of the pulmonary interstitium [3]. The underlying mechanism of lung injury is believed to involve alveolar macrophages. Silica is cytotoxic to macrophages; if macrophages survive the initial exposure, they engulf the particles and migrate into the interstitium. Once internalized, silica enters the lysosomes, which lack the enzymes to degrade it. This leads to lysosomal membrane rupture, the release of lysosomal enzymes, and the production of reactive oxygen species (ROS). ROS trigger caspase activation, leading to the release of pro-inflammatory and fibrotic mediators such as tumor necrosis factor alpha, transforming growth factor beta (TGF-β), and platelet-derived growth factor, which drive apoptosis and tissue damage. The cycle of macrophage apoptosis and silica release perpetuates the process, ultimately resulting in pulmonary fibrosis [2].

In 2017, a total of 23,695 cases of silicosis were reported worldwide [5]. Despite being a serious public health concern, silicosis currently has no definitive treatment. Efforts have thus focused on prevention, although protective measures are not always implemented by employers or consistently followed by workers. Individuals with silicosis face an increased risk of tuberculosis, bronchogenic carcinoma, autoimmune disorders, and mortality from non-carcinogenic lung diseases [6].

We report the case of a 50-year-old otherwise healthy male who presented with dyspnea and was subsequently diagnosed with silicosis.

## Case presentation

A 50-year-old male with no known past medical history presented to our hospital with dyspnea that had been ongoing for 10 months and had worsened over the preceding four days. His symptoms were accompanied by a dry cough, fatigue, and poor appetite, although he denied weight loss. He also reported experiencing a sore throat, rhinorrhea, subjective fevers (not measured at home), and intermittent headaches. He denied chest pain, back pain, abdominal pain, nausea, vomiting, diarrhea, dysuria, dizziness, or generalized weakness.

The patient had moved to the United States from Mexico approximately 33 years ago, with his last visit to Mexico occurring around 14 years prior. He denied any other travel history or contact with sick individuals. Notably, he reported working in a granite factory for the past 18 years, primarily engaged in grinding and cutting stone. He denied any history of prior pulmonary infections, tuberculosis, childhood asthma, tobacco use, or smoking. He also stated that he had not received any vaccinations since immigrating to the United States. A purified protein derivative test performed four months earlier had been negative.

**Figure 1:**
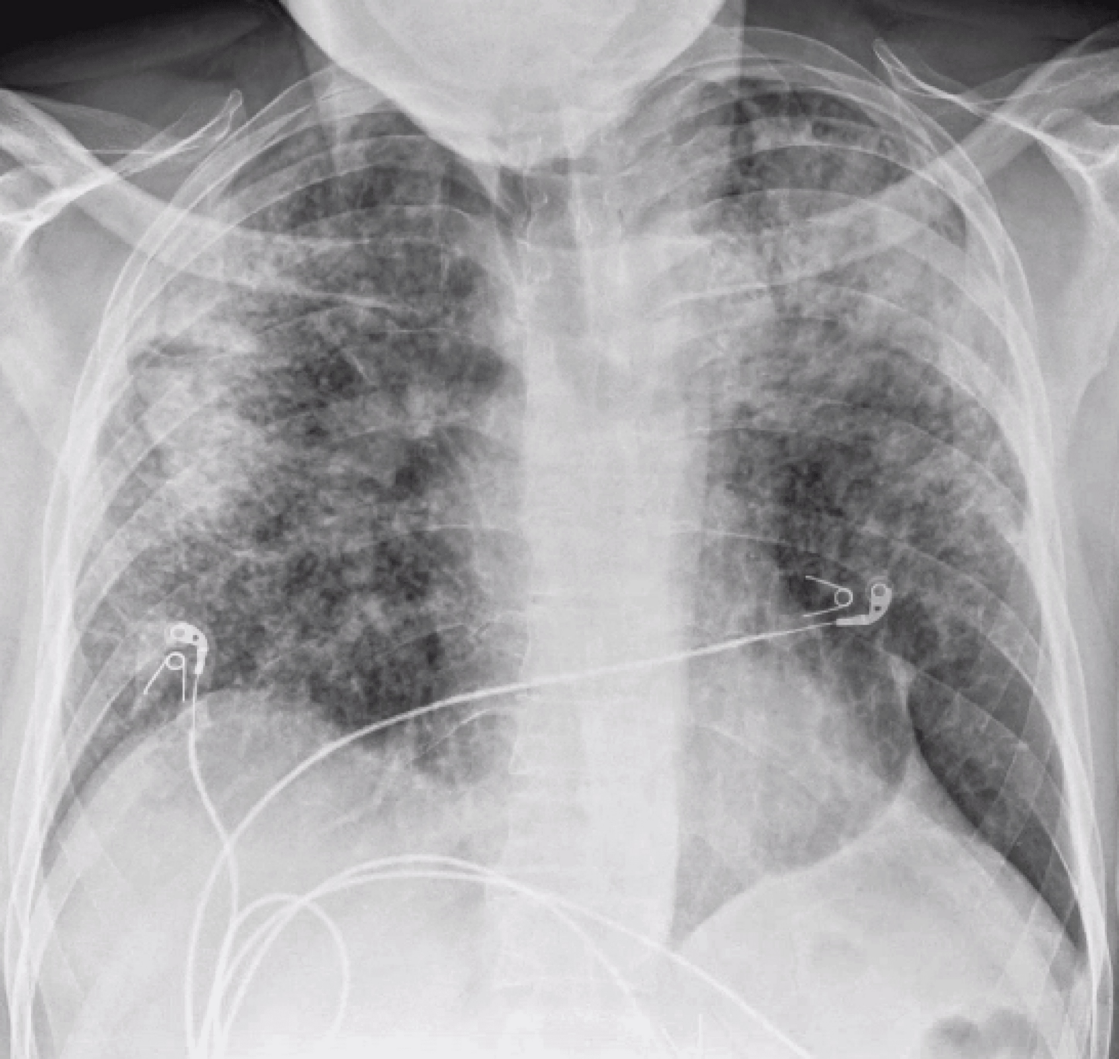
CXR at admission CXR, chest radiograph

**Figure 2:**
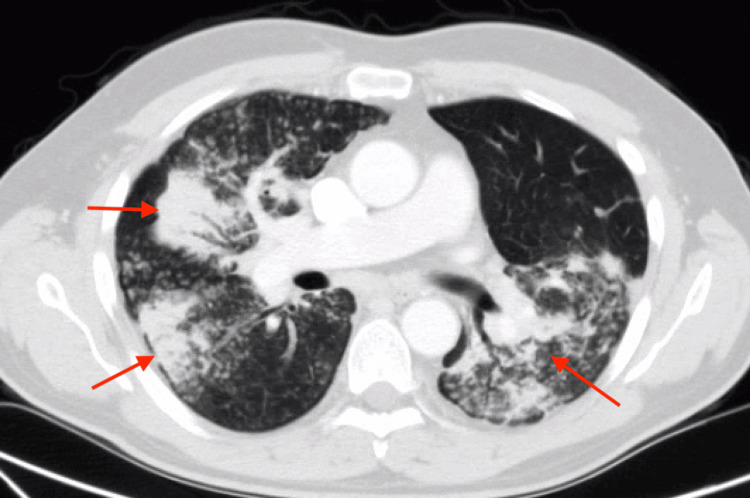
Transverse CT chest view at admission

**Figure 3:**
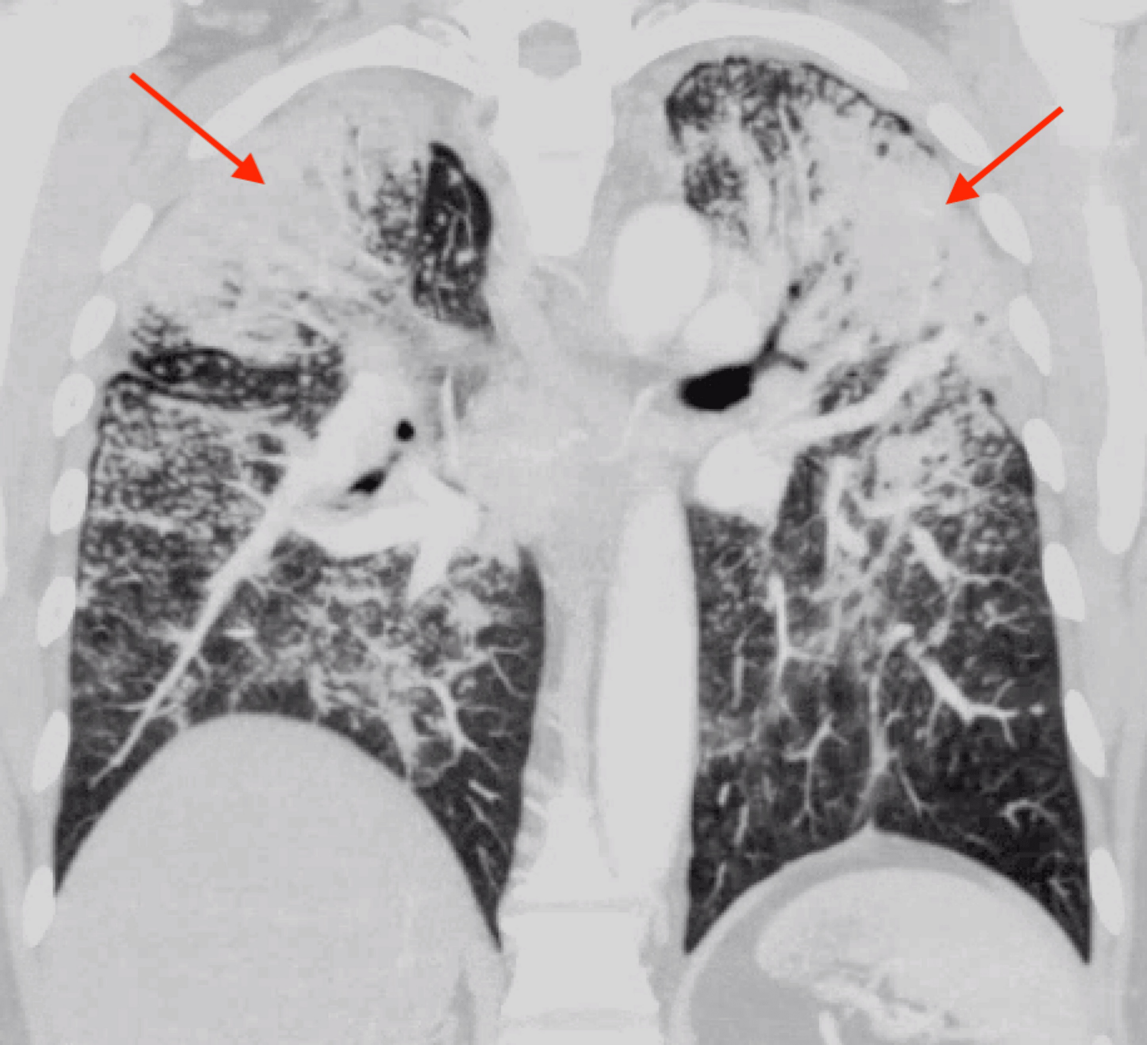
Coronal CT chest view at admission

On presentation, he was hemodynamically stable and afebrile. His oxygen saturation was 98% on room air, and his respiratory rate was 16 breaths per minute. On physical examination, pulmonary effort was normal, and breath sounds were clear bilaterally without rales, wheezes, or crackles. Laboratory values on admission are listed in Table 1. Chest radiograph (CXR) revealed bilateral patchy opacities, particularly prominent in the upper lung zones (Figure 1). A CT scan of the chest with IV contrast (Figure 2, Figure 3) demonstrated extensive bilateral pulmonary nodules with patchy areas of consolidation in the upper lobes, along with enlarged mediastinal lymph nodes. CT imaging of the abdomen and pelvis was non-contributory.

**Table 1:** Laboratory values at admission

Parameter	Laboratory value	Reference range
Complete blood count		
White blood cell	7.6 × 10³/µL	4.5-11.1 × 10³/µL
Hemoglobin	14.6 g/dL	13.0-17.0 g/dL
Hematocrit	44%	41-53%
Platelets	546 × 10³/µL	120-360 × 10³/µL
Basic metabolic panel		
Sodium	140 mmol/L	135-148 mmol/L
Potassium	4.6 mmol/L	3.5-5.5 mmol/L
Chloride	107 mmol/L	98-110 mmol/L
Bicarbonate	20 mmol/L	24-34 mmol/L
Blood urea nitrogen	10 mg/dL	8-20 mg/dL
Creatinine	0.92 mg/dL	0.50-1.50 mg/dL
Glucose	98 mg/dL	65-125 mg/dL
Calcium	9.2 mg/dL	8.5-10.5 mg/dL
Respiratory viral panel		
Adenovirus	Not detected	Not detected
Coronavirus 229E	Not detected	Not detected
Coronavirus HKU1	Not detected	Not detected
Coronavirus NL63	Not detected	Not detected
Coronavirus OC43	Not detected	Not detected
SARS-CoV-2	Not detected	Not detected
Human metapneumovirus	Not detected	Not detected
Human rhinovirus/enterovirus	Not detected	Not detected
Influenza A	Not detected	Not detected
Influenza B	Not detected	Not detected
Parainfluenza 1	Not detected	Not detected
Parainfluenza 2	Not detected	Not detected
Parainfluenza 3	Not detected	Not detected
Parainfluenza 4	Not detected	Not detected
Respiratory syncytial virus	Not detected	Not detected
* Bordetella parapertussis*	Not detected	Not detected
* Bordetella pertussis*	Not detected	Not detected
* Chlamydia pneumoniae*	Not detected	Not detected
* Mycoplasma pneumoniae*	Not detected	Not detected

He was initially placed on airborne isolation, and sputum samples were collected for acid-fast bacilli (AFB) smear and culture to rule out tuberculosis. A respiratory viral panel was negative. Empiric treatment for community-acquired pneumonia was initiated with azithromycin 500 mg orally once daily and ceftriaxone 2 g IV once daily. A 500 mL bolus of normal saline was also administered. Pulmonology was consulted and recommended further fungal studies, including coccidioides, *Histoplasma*, and blastomyces antibodies, beta-D-glucan, as well as an interferon-γ release assay. Urinary antigens for *Legionella *and *Streptococcus pneumoniae *were also ordered.

On hospital day 3, the patient developed leukocytosis, with a white blood cell (WBC) count rising to 17.3 × 10³/μL. Respiratory cultures grew normal respiratory flora, including gram-positive cocci in pairs, chains, and clusters. Repeat respiratory cultures were obtained, and due to the worsening leukocytosis, antibiotic coverage was broadened to include pseudomonal coverage - ceftriaxone was replaced with cefepime 2 g IV every eight hours. After three doses of azithromycin for atypical coverage were completed, the drug was discontinued, given the absence of atypical organisms on culture.

Three AFB smears showed no AFB, and *Mycobacterium tuberculosis *testing was negative, leading to the discontinuation of airborne isolation precautions. The WBC count normalized within two days of starting cefepime. Urinary antigens for *Legionella *and *S. pneumoniae *returned negative. All fungal studies were negative except for the *Histoplasma* antibody complement fixation test, which detected a 1:16 yeast phase antibody and a 1:8 mycelial phase antibody; however, the *Histoplasma *urine antigen test was negative.

**Figure 4:**
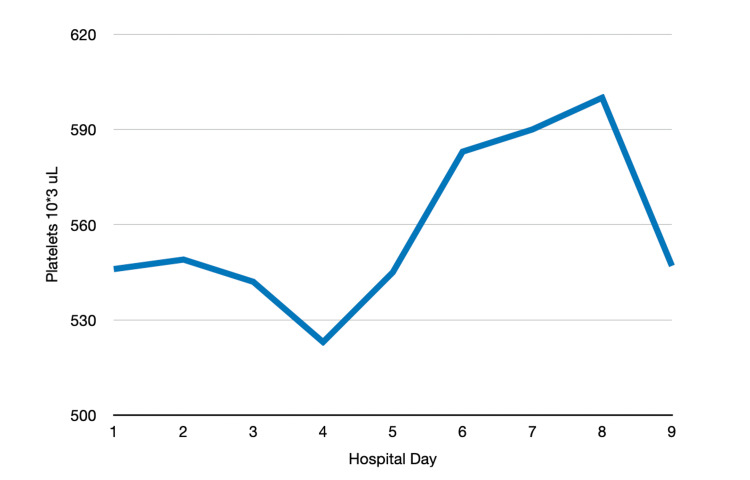
Platelet trend throughout hospitalization

Given the inconclusive results, bronchoscopy with bronchoalveolar lavage (BAL) was performed on hospital day 8 in the right middle lobe and left lingula. Throughout the hospital stay, the patient remained afebrile and maintained stable vital signs, despite reporting intermittent subjective fevers. Thrombocytosis persisted during the hospitalization (Figure 4). The patient was discharged in stable condition with plans for outpatient follow-up with pulmonology and a repeat chest CT in four weeks.

The patient was seen in the pulmonology clinic after discharge. Cytology from BAL samples of the right middle lobe and left upper lobe revealed numerous foamy macrophages and intracellular crystalline spicules under polariscopy, findings suggestive of silicosis. There was no evidence of malignancy, fungal infection, or acute bacterial infection.

The following tests from the BAL of both the right middle and left upper lobes were unremarkable: *Legionella *culture, cytomegalovirus culture, herpes simplex virus culture, *Pneumocystis jirovecii *direct fluorescence antibody, AFB smear and culture, *Mycoplasma pneumoniae *DNA PCR, and *Coccidioides *antigen enzyme immunoassay. Fungal culture of the left upper lobe BAL was negative, while that of the right middle lobe grew mold later identified as *Penicillium *species, likely a contaminant. Remaining significant laboratory values are presented in Table 2.

**Table 2:** BAL results BAL, bronchoalveolar lavage

Test	Result	Reference range
BAL cell differential – left upper lobe		
Polymorphonuclear leukocytes	6%	2-11%
Lymphocytes	46%	2-25%
Macrophage	40%	60-80%
Bronchial epithelial cells	8%	3-21%
BAL cell differential – right middle lobe		
Polymorphonuclear leukocytes	6%	2-11%
Lymphocytes	56%	2-25%
Macrophage	33%	60-80%
Bronchial epithelial cells	5%	3-21%

A repeat chest CT showed no significant interval change in the bilateral patchy consolidations and nodules, with continued prominence of mediastinal lymph nodes, compared to the imaging three months prior. Based on the BAL findings and absence of alternative explanations for the patient’s dyspnea, a diagnosis of anthracosilicosis was made.

The patient was also seen in the infectious diseases clinic after discharge. Repeat *Histoplasma *antibody complement fixation again detected a 1:16 yeast phase antibody and a 1:8 mycelial phase antibody. However, a quantitative *Histoplasma *antigen test showed no antigen detected. Given the low clinical suspicion for true histoplasmosis, antifungal therapy was not initiated.

## Discussion

The pathogenesis of silicosis is primarily mediated by alveolar macrophages. When these cells engulf silica particles, they trigger a cascade of lymphocyte and leukocyte recruitment, leading to the release of factors that stimulate collagen deposition and hyalinization [2,3]. The end result is the formation of a nodular lesion around the initial site of silica deposition. This process, as mentioned earlier, also involves lysosomal rupture and macrophage apoptosis. The patient’s BAL findings align with these pathogenic mechanisms, showing an increase in lymphocytes and a decrease in macrophages, likely due to macrophage cell death.

Silicosis has also been linked to enhanced peripheral thrombosis, a phenomenon observed in our patient [7]. In an animal model, Nemmar et al. proposed that pulmonary macrophages activate neutrophils, which then release neutrophil elastase, triggering platelet activation. Inhibiting neutrophil elastase in this model led to a reduction in peripheral thrombogenicity [7]. The brief leukocytosis observed early in our patient’s hospitalization was most likely reactive, as the patient did not meet the criteria for systemic inflammatory response syndrome and was never septic.

Initially, the patient’s pulmonary lesions, subjective fever, and upper respiratory symptoms raised concerns about infection. Patients with silicosis are known to be at higher risk for mycobacterial and fungal infections, particularly pulmonary tuberculosis. In fact, *M. tuberculosis *accounts for approximately half of all infections superimposed on silicosis [6]. However, in this case, all subsequent fungal and mycobacterial tests were negative, except for *Histoplasma *complement fixation. It is important to note that *Histoplasma *complement fixation has a false-positive rate of up to 35%, which likely explains the positive result in our patient, as the quantitative *Histoplasma *antigen test was negative [8].

The pathognomonic chest X-ray findings in silicosis typically show numerous pulmonary nodules, each less than 10 mm in diameter, bilaterally. These nodules are generally round in shape and predominantly distributed in the upper lobes of the lungs [4]. However, chest X-ray has been shown to have a poorer correlation with functional pulmonary impairment compared to high-resolution CT (HRCT). HRCT is not routinely indicated for silicosis patients unless they present with atypical clinical or radiographic features [4,9]. Additionally, HRCT reduces reader-to-reader variability and is better at detecting changes, coalescence, and conglomeration of nodules [4,9].

Silicosis is not just a patient-specific issue but a significant public health concern that requires a systemic approach. To standardize screening for silicosis, the International Labor Organization (ILO) has developed a classification system for pneumoconioses based on CXR. This classification takes into account the technical quality of the image, parenchymal opacities, pleural thickening, and costophrenic angle obliteration. The specificity of the ILO classification for diagnosing silicosis ranges from 71% to 100% [4]. Both the ILO and WHO have established the Global Programme for the Elimination of Silicosis, under which countries are required to implement national programs for the elimination of silicosis (NPES). Countries with active NPES include Brazil, Chile, China, India, Peru, South Africa, Thailand, Turkey, and Vietnam. While silicosis mortality has decreased over the past few decades, this decline is less pronounced worldwide, with countries like Palau, North Korea, Chile, China, and Portugal bearing the highest burden. In the US, the issue remains that Black workers are two to seven times more likely to develop silicosis than White workers when exposed to inhaled silica, suggesting an additional influence of social determinants of health [4].

Silicosis has also been associated with autoimmune diseases, such as myositis and progressive systemic sclerosis (PSS). The immunological pathogenesis of silicosis is complex and multifactorial, with mixed effects. On one hand, there appears to be a positive correlation between silicosis and autoimmune diseases. On the other hand, there is also evidence of suppressed immune responses due to silica exposure. One of the key players in the immunological effects of silicosis is the Fas/Fas ligand (FasL) system, which includes soluble Fas (sFas).

In both in vivo and in vitro studies, silica exposure leads to increased FasL expression in macrophages, which promotes FasL-mediated macrophage apoptosis. The release of pro-inflammatory markers from silica-exposed macrophages further determines whether FasL’s effects are suppressive or pro-inflammatory, with TGF-β playing a significant role. In most cases, FasL has an inflammatory effect. For example, anti-FasL antibodies that block FasL function have been shown to prevent the development of silicosis in vivo. Conversely, artificially induced FasL expression in tumors and grafts accelerates rejection by triggering a neutrophil-mediated inflammatory pathway [10].

Silicosis activates a chain of complex molecular pathways. As mentioned, silica induces macrophage apoptosis by increasing FasL expression. Additionally, silica exposure also raises levels of sFas, which antagonizes FasL in Fas/FasL interactions, similar to the function of anti-FasL antibodies [11]. This antagonism of FasL has been proposed to suppress T cell apoptosis and activate T cells, potentially increasing the risk of autoimmune disease development [12]. However, other studies have not supported this theory. In vivo studies have shown that the introduction of silica particles into the trachea or intravenously is associated with increased skin allograft and bone marrow graft survival and a suppression of T cell function [13].

PSS, also known as Erasmus syndrome, was first described in 1957 as a rare immune-mediated connective tissue disease in which systemic sclerosis (SSc) develops following silica exposure, even in the absence of silicosis [14]. One proposed mechanism of this pathogenesis involves silica-induced dysregulation of T lymphocytes. In a study by Magnant et al., SSc patients previously exposed to silica were more likely to present with diffuse cutaneous SSc, digital ulcers, and interstitial lung disease [15]. However, other studies found no significant differences in the clinical presentation between SSc patients exposed to silica and those not exposed [13]. Myositis, an inflammatory autoimmune disease of the musculoskeletal system, has also been linked to silica exposure. In a study by Parks et al., moderate to high silica exposure was associated with increased systemic autoimmune rheumatic disease-associated myositis in both smokers and nonsmokers [16].

Currently, there is no available treatment for silicosis. Our patient reported wearing a mask during work; however, it remains unclear whether the mask was properly fitted as a true respirator, whether it was in good condition with unclogged filters, and whether the patient consistently used it properly, ensuring no air leaks. As for workplace regulations, Garcia et al. suggest several preventive measures, including drilling with simultaneous water application, preventing ore from falling from great heights, misting sites after detonations, and utilizing environmental ventilation with bag filters [1]. Further recommendations include the development of national policies and university specialization to create a workforce division dedicated to environmental safety, especially in countries that currently lack such programs and regulatory bodies.

Ongoing research into treatments for silicosis includes anti-fibrotic agents, anti-cytokine therapies, agents targeting autophagy-lysosome systems, antioxidants, agents that increase cyclic adenosine monophosphate (cAMP), and those that affect TGF-β. A 2015 case published in the American Thoracic Society explored the use of anakinra to inhibit IL-1β in a patient with silicosis after seven years of exposure [17]. The patient underwent daily treatment for six months, showing improvements in functional outcomes, including increased partial pressure of oxygen in arterial blood, improved diffusion capacity of the lungs for carbon monoxide, and decreased tracer uptake in lung parenchyma on a PET scan [17]. However, without any FDA-approved treatments for silicosis at present, preventive measures, particularly those implemented and enforced at a systemic level, remain crucial.

## Conclusions

Silicosis should be suspected in patients with a history of working in industries such as quarrying, stone cutting, construction, and sandblasting. HRCT of the chest is recommended if the chest X-ray shows abnormalities, as it offers better correlation with functional pulmonary impairment, improved lesion detection, and reduced reader-to-reader variability. Currently, there are no FDA-approved treatments for silicosis. However, investigational therapies, including anti-fibrotic agents, anti-cytokine treatments, agents targeting autophagy-lysosome systems, antioxidants, cAMP-increasing agents, and TGF-β modulators, are being studied.

Silicosis is a systemic issue that requires a broader, public health approach rather than just focusing on individual patient care. Given the lack of effective treatments, preventative measures are crucial. Recommended workplace interventions include drilling with simultaneous water application, preventing ore from falling from significant heights, misting sites after detonations, and employing environmental ventilation with bag filters. Additionally, respirator use should be enforced with proper training, fitting, and regular supply checks.
